# P-1095. Activity of Rezafungin against Clinical Isolates of Uncommon Species of *Candida spp*

**DOI:** 10.1093/ofid/ofae631.1283

**Published:** 2025-01-29

**Authors:** Marisa Winkler, Paul Rhomberg, Abigail Klauer, Mariana Castanheira

**Affiliations:** Element Materials Technology/Jones Microbiology Institute, North Liberty, Iowa; Element Materials Technology/Jones Microbiology Institute, North Liberty, Iowa; Element Materials Technology/Jones Microbiology Institute, North Liberty, Iowa; JMI Laboratories, North Liberty, Iowa

## Abstract

**Background:**

Rezafungin (RZF) is a long-acting echinocandin recently approved by the FDA to treat invasive candidiasis (IC) and candidemia. RZF CLSI breakpoints are available for *Candida albicans*, *C. auris* (CARS), *C. dubliniensis* (CDUB), *C. glabrata*, *C. krusei*, *C. parapsilosis* (CPRP), and *C. tropicalis*. However, RZF susceptibility results are scarce against other *Candida* spp. We evaluated *in vitro* activity of RZF, anidulafungin (AND), caspofungin (CAS), micafungin (MCF), fluconazole (FLC), voriconazole (VRC), and amphotericin B (AMB) against less common species of *Candida* spp. including CARS and CDUB.

**Figure:**
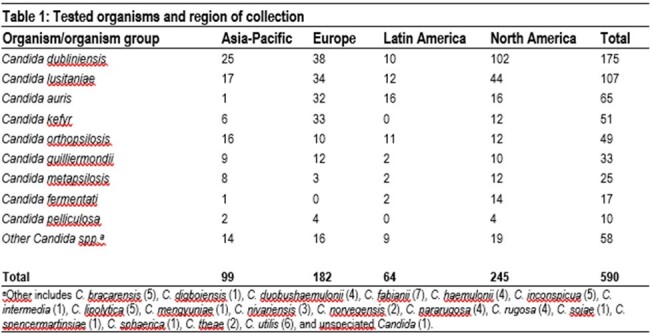

**Methods:**

We tested 590 (1/patient) less common *Candida* spp. isolates from IC infections as part of the SENTRY Program (Table 1). Isolates were collected from 2020-2022 in 80 hospitals including 31 in North America, 29 in Europe, 14 in Asia-Pacific, and 6 in Latin America. Isolates were identified by MALDI-TOF MS and/or sequencing and susceptibility testing was performed using the CLSI broth microdilution method. CLSI breakpoints or epidemiologic cutoff values were applied where available.

**Figure:**
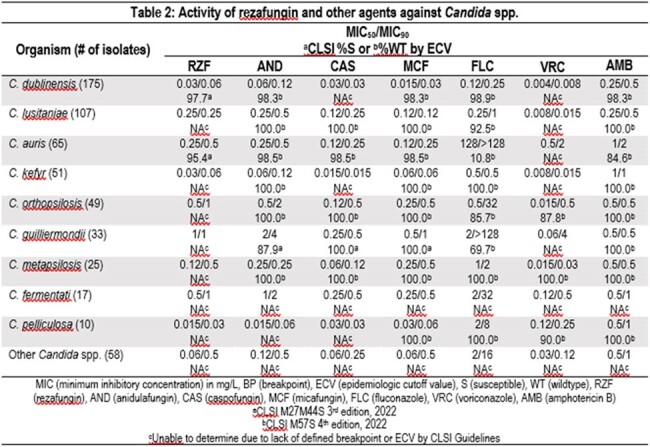

**Results:**

RZF displayed similar activity to other echinocandins (within ±2-fold for MIC_50_/_90_ values) against all *Candida* spp. tested (Table 2). The lowest RZF MIC_50_/_90_ values were noted against *C. kefyr* (n=51, 0.03/0.06 mg/L) and *C. pelliculosa* (n=10, 0.015/0.03 mg/L). Higher RZF MIC_50/90_ values were noted for *C. guilliermondii* (CGU, n=33, 1/1 mg/L) and for isolates in the CPRP complex (*C. orthopsilosis* (CORT) n=49, 0.5/1 mg/L*, C. metapsilosis* (CMET) n=25, 0.12/0.5 mg/L). The same pattern was seen with other echinocandins against these *Candida* spp. RZF was active against 97.7% of CDUB and 95.4% of CARS isolates applying the CLSI breakpoints. There were three CARS isolates that were resistant to RZF with MICs of 1- >4 mg/L. Two of these were susceptible to CAS and one was resistant or non-wildtype to all echinocandins. For FLC 69.7% of CGU, 85.7% of CORT, 100% CMET, and only 10.8% of CARS isolates were susceptible.

**Conclusion:**

RZF has potent *in vitro* activity against a large collection of less common *Candida* spp. as demonstrated by worldwide surveillance testing. The activity of RZF against these isolates was comparable to other echinocandins and generally better than FLC.

**Disclosures:**

**Marisa Winkler, MD, PhD**, Element Iowa City (JMI Laboratories) was contracted to perform services in 2023 for > 30 biotech and pharmaceutical companies: Grant/Research Support

